# A Non-Invasive Interpretable Diagnosis of Melanoma Skin Cancer Using Deep Learning and Ensemble Stacking of Machine Learning Models

**DOI:** 10.3390/diagnostics12030726

**Published:** 2022-03-17

**Authors:** Iftiaz A. Alfi, Md. Mahfuzur Rahman, Mohammad Shorfuzzaman, Amril Nazir

**Affiliations:** 1 Department of Electrical and Computer Engineering, North South University, Dhaka 1229, Bangladesh; iftiaz.alfi@northsouth.edu; 2 Department of Information and Computer Science, College of Computing and Mathematics, King Fahd University of Petroleum and Minerals, Dhahran 31261, Saudi Arabia; mdmahfuzur.rahman@kfupm.edu.sa; 3 Interdisciplinary Research Center for Intelligent Secure Systems, King Fahd University of Petroleum and Minerals, Dhahran 31261, Saudi Arabia; 4 Department of Computer Science, College of Computers and Information Technology, Taif University, Taif 21944, Saudi Arabia; 5 Department of Information Systems, College of Technological Innovation, Abu Dhabi Campus, Zayed University, Abu Dhabi P.O. Box 144534, United Arab Emirates; mohd.nazir@zu.ac.ae

**Keywords:** skin cancer, diagnosis, machine learning, stacking model, deep learning, interpretability, melanoma

## Abstract

A skin lesion is a portion of skin that observes abnormal growth compared to other areas of the skin. The ISIC 2018 lesion dataset has seven classes. A miniature dataset version of it is also available with only two classes: malignant and benign. Malignant tumors are tumors that are cancerous, and benign tumors are non-cancerous. Malignant tumors have the ability to multiply and spread throughout the body at a much faster rate. The early detection of the cancerous skin lesion is crucial for the survival of the patient. Deep learning models and machine learning models play an essential role in the detection of skin lesions. Still, due to image occlusions and imbalanced datasets, the accuracies have been compromised so far. In this paper, we introduce an interpretable method for the non-invasive diagnosis of melanoma skin cancer using deep learning and ensemble stacking of machine learning models. The dataset used to train the classifier models contains balanced images of benign and malignant skin moles. Hand-crafted features are used to train the base models (logistic regression, SVM, random forest, KNN, and gradient boosting machine) of machine learning. The prediction of these base models was used to train level one model stacking using cross-validation on the training set. Deep learning models (MobileNet, Xception, ResNet50, ResNet50V2, and DenseNet121) were used for transfer learning, and were already pre-trained on ImageNet data. The classifier was evaluated for each model. The deep learning models were then ensembled with different combinations of models and assessed. Furthermore, shapely adaptive explanations are used to construct an interpretability approach that generates heatmaps to identify the parts of an image that are most suggestive of the illness. This allows dermatologists to understand the results of our model in a way that makes sense to them. For evaluation, we calculated the accuracy, F1-score, Cohen’s kappa, confusion matrix, and ROC curves and identified the best model for classifying skin lesions.

## 1. Introduction

Skin is the largest organ of the human body. It is the only organ that is in constant contact with the external environment. The skin keeps the internal temperature of the body consistent and protects from harmful agents entering the body. Since the skin directly interacts with the environment, many factors affect it significantly, such as friction, pressure, vibration, heat, cold, radiation, virus, bacteria, insects, etc., and a variety of other chemicals.

As the skin is at constant exposure, the chance of obtaining a skin disease is higher. Malignant skin cancer, commonly known as malignant melanoma, is one of the most dangerous types of skin cancer. It is caused by long-term UV exposure, which induces mutations in melanocytes, the cells that make melanin pigment [1]. When exposed to UV radiation, melanocytic cells create an excessive amount of melanin. As a result, black moles appear on the skin. These moles on the skin can grow into malignant tumors that spread fast to other parts of the body. Melanoma is one of Australia’s most prevalent malignancies. The top cancers contribute to approximately 60% of total cancers diagnosed in the country [2]. In Australia, an estimated 434,000 persons were diagnosed with one or more non-melanoma skin malignancy in 2008. A total of 679 Australians died in 2016 from non-melanoma skin cancer. According to a report of the American Cancer Society [3], in 2021, 6% of all diagnosed cancers were melanoma. Melanoma affects one out of every twenty-seven males and one out of every forty females. In 2021, the United States saw 1.9 million new cancer diagnoses and 608,570 cancer deaths. Melanoma cases are very high in sun-exposed areas. By 2020, melanoma is anticipated to be Australia’s third most common cancer, and New Zealand has the highest melanoma incidence rate in the world. In Australia, 16,878 new cases of melanoma are predicted to be discovered in 2021.

**Figure 1:**
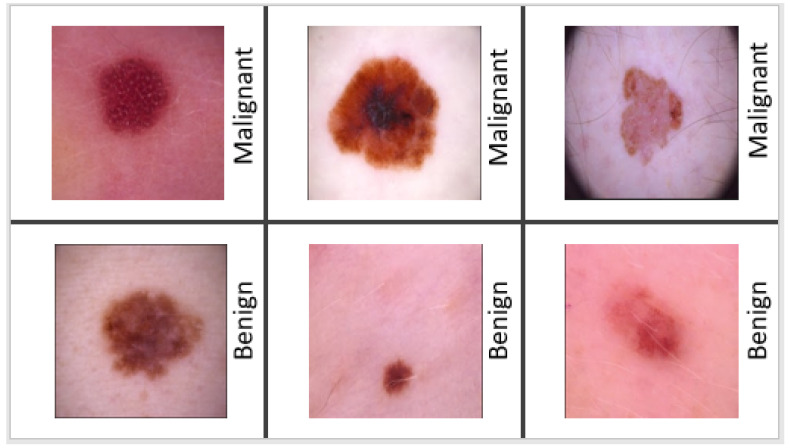
Example of malignant and benign skin lesions.

Melanoma may appear in a variety of ways. The initial indicator is generally the appearance of a new mole or a change in an existing mole. The dermatologist typically applies the “ABCDE” criterion to diagnose skin cancer: (i) A—Asymmetry (irregular) (ii) B—Border (uneven or scalloped edges), (iii) C—Colour (differing shades and color patches), (iv) D—Diameter (usually over 6mm), (v) E—Evolving (changing and growing). After applying the “ABCDE” criterion, they decide whether the mole is malignant or benign. If the mole is suspected of melanoma, a skin biopsy or other methods are performed for confirmation of melanoma. If it is a benign mole, then the patient needs time to time the “ABCDE” assessment in order to keep track of its condition. In the United States, the 5-year survival rate is 98 percent, but it drops to 18 percent after cancer spreads to distant organs. As a result, the early identification of melanoma is critical for increasing survival rates. Malignant melanoma can be treated if diagnosed early enough [4,5] according to experimental research. Figure 1 displays examples of a collection of malignant and benign skin lesions.

It can be difficult to recognize melanoma from all melanoma photos because of the variety of features, such as a low contrast, noise, and uneven borders. Moreover, ABCD criteria are not always reliable in determining whether or not an individual has skin cancer, as per the research findings [6]. Additionally, these procedures require highly trained dermatologists to minimize the possibility of making an incorrect diagnosis, which results in a high rate of false positive and negative instances. To overcome these limitations, this article has focused on developing a deep-learning-based computer-aided method for analyzing lesions on skin to detect potential skin cancer.

Automated melanoma detection algorithms also began to appear in the literature, giving timely services to dermatologists of all levels. Two approaches based on statistical learning are widely used. Firstly, standard classification methods rely on skin lesion features that are typically difficult to generate and require a specific understanding of the domain. The second category of methods are based on deep learning models that can automatically extract disease-related features for skin lesion classification. Nonetheless, accuracies have been hampered thus far due to image occlusions and imbalanced datasets. While deep learning algorithms have demonstrated their superiority to conventional strategies for melanoma identification, the majority of these approaches lack an adequate explainability of the models associated with relevant aspects of pathological symptoms. As a result, the clinical usefulness of these strategies is unknown until more research is conducted to understand the high-level properties collected from these models. Even with highly precise experimental results, dermatologists are unlikely to adopt a black-box categorization approach in the real world.

Hence, the overall objective of this research is to introduce an interpretable method for non-invasive diagnosis melanoma skin cancer using deep learning and ensemble stacking of machine learning models. More specifically, the proposed research has the following four objectives to be achieved: (i) to build a stacked ensemble of various machine models that are trained using hand-crafted features extracted from melanoma images for a non-invasive diagnosis of melanoma skin cancer; (ii) to create an ensemble of deep learning models that are fine-tuned with automated features extracted from melanoma images; (iii) to assess the effectiveness of our proposed models using a publicly available skin lesion dataset over a wide range of parameters; (iv) to construct an interpretability approach that generates heatmaps to identify the parts of a melanoma image that are most suggestive of the illness.

Our statistical hypothesis testing was carried out using the corrected paired Student’s *t*-test to see whether the differences in the performances of these models were statistically significant or not [7]. To accomplish this, the paired *t*-test is utilized using the following null and alternative hypotheses: H0, no difference in performance exists between the two deep learning models when comparing them; HA, the two deep learning models exhibit significant differences in performance.

In this paper, we proposed an interpretable approach to melanoma skin cancer diagnosis based on deep learning and ensemble stacking of machine learning models. Hand-crafted features based on shape, texture, and color were used to train the base machine learning models, namely, logistic regression, support vector machine (SVM), random forest, k-nearest neighbor (KNN), and gradient boosting machine. The prediction of these base models was used to train level one model stacking [8,9] using cross-validation on the training set. Deep learning models (MobileNet, Xception, ResNet50, ResNet50V2, and DenseNet121) were used for transfer learning, and were already pre-trained on ImageNet data. The selected base models were trained and tested on a small dataset [10] of the ISIC 2018 challenge [11], and the results of each model were compared. The best models were then ensembled to increase the accuracy. The dataset contains only two classes: malignant and benign. However, the methods are also capable of classifying the dataset with more categories. Furthermore, shapely adaptive explanations were used to construct an interpretability approach that generates heatmaps to identify the parts of a melanoma image that are most suggestive of the illness. Our method shows promising results in diagnosing melanoma skin cancer, leading to clinical advantages. To summarize, we have made the following significant contributions in this paper:

- Feature extractions were performed using Hu moments, Haralick features, and a color histogram;
- Transfer learning was used by fine-tuning the backbones, and the base models were pre-trained on ImageNet data;
- A performance comparison was carried out between machine learning and deep learning models;
- Level one stacking was performed on machine learning models;
- Ensembling was carried out from different combinations of deep learning models;
- Extensive experimentation was conducted to illustrate the model’s performance, with a final accuracy of 92.0% and an AUC score of 97.0% for skin lesion classification.

We structure the rest of the paper as follows. Section 2 includes the related work. Section 3 describes the dataset used and the model architecture of our two methods. Section 4 elaborately explains the graphs and results of the models. Finally, Section 5 concludes the paper with an idea of future work.

## 2. Related Work

This section provides a brief description of the related works that focus on skin lesion classifications. It describes different techniques and approaches followed and addresses skin lesion classifications’ common problems and their solutions.

### 2.1. Towards Automated Melanoma Detection with Deep Learning: Data Purification and Augmentation

Devansh et al. [12] proposed two main problems with the public dataset: (a) the datasets are small and imbalanced and (b) the dermoscopic images contain occlusions. The paper mainly focuses on the dermoscopic images because of their higher diagnostic accuracy. The problems mentioned need to be solved. Therefore, they proposed a method of data purification to remove the occlusion and used de-coupled DCGANs to generate new melanoma and seborrheic keratosis class images with data augmentation to balance the dataset. The ROC-AUC value that was achieved for the melanoma class with their proposed method was 0.880, whereas the ISIC challenge winners was 0.874, and so the performance improved by 4%. In another study by Esteva et al. [13], two dermatologists achieved 65.56% and 66%, and their model obtained an accuracy of 81.6%.

### 2.2. Melanoma Skin Cancer Detection Using Image Processing and Machine Learning

The paper [14] proposed a method where hair, shading, and glares are removed from the images in the pre-processing part. Then, segmentation and feature extraction are carried out. In the last stage of their method, they trained their model on the back propagation algorithm (feed-forward neural network), SVM, and CNN. After classification, the models were amalgamated (combined) with image processing tools, leading to an accuracy of 85% on the ISIC dataset.

### 2.3. Melanoma Skin Cancer Detection Using Deep Learning and Classical Machine Learning Techniques: A Hybrid Approach

Daghrir et al. [15] offered a strategy that relies on predictions from three separate methods: CNN and two machine learning classifiers. A collection of features characterizing the texture, boundaries, and color of the skin lesion is used to train the classifiers. These strategies are then integrated using a majority vote to increase the performance. The experiment was carried out using a publicly available dataset from the ISIC repository. They dealt with just 640 photos, including benign and malignant lesions, out of 23,000 melanoma images. The three approaches of KNN, SVM, and CNN attained an accuracy of 57.3%, 71.8%, and 85.5%, respectively, according to their findings. The accuracy increased to 88.4% after the majority vote. The research also raises certain concerns, such as a shortage of labeled data on which to train the system, which is considered to be addressed through semi-supervised learning. The majority of systems are based on two rules: ABCD and the blue-black rule, both of which have flaws and have proven to be ineffectual in various circumstances. As a result, a new idea called ’ugly duckling’ was presented, in which, researchers looked at not only the morphology of the lesion in issue but also compared it to that of other lesions, seeking an outlier amid a sea of similar-looking moles. In a subsequent effort, Filali et al. [16] proposed a skin lesion classification method by performing a fusion of hand-crafted features (e.g., shape, texture, color) and automated features extracted from deep CNN models. The model was trained and tested with a small (PH2) and large (ISIC challenge) dataset. The experimental results demonstrated a better classification performance using the PH2 dataset.

Mahbod et al. [17] suggested an autonomous computerized technique for skin lesion categorization that makes use of deep characteristics derived from convolutional neural networks. They used three deep models that have been pre-trained. The derived features were utilized to train SVM classifiers with the ISIC challenge dataset, which achieved an accuracy rate of 83%. Amin et al. [18] proposed an integrated method of deep feature fusion to detect skin cancer. Relevant features are extracted from the segmented skin lesion images using AlexNet and VGG-16 and are then fused in order to be used in the classification task. PCA was used to select the optimal feature set. A curated dataset was used for model training and validation.

### 2.4. Melanoma Detection by Analysis of Clinical Images Using Convolutional Neural Network

Nasr et al. [19] proposed a method to classify skin diseases between malignant and benign from non-dermoscopic skin images. In the pre-processing part, the illumination is corrected and mask generated, and a gaussian filter is applied to smooth the surface of the normal skin part. The limitation of images is dealt with by rotating, cropping, and resizing the photos. Then, the images are split into an 80:20 ratio. The training set is then fed into a CNN model, and the metrics calculation is performed. Compared with other methods, it gave an accuracy of 81.0%, where the second-best model (MED-NODE texture descriptor [20]) had an accuracy of 76.0%. In another effort, Bajwa et al. [21] utilized deep neural network for the classification of hundreds of skin diseases while improving the classification performance. They used two popular skin lesion datasets, called DermNet and ISIC, and achieved an accuracy of 80% and 93%, respectively. Moreover, Bi et al. [22] presented a hyper-connected CNN model for multi-modal skin lesion classification. Their approach is capable of producing stable classification results, even when the class distributions are unbalanced.

### 2.5. An Efficient 3D Color-Texture Feature and Neural Network Technique for Melanoma Detection

Warsi et al. [23] reported an image enhancement, segmentation, and classification approach based on D-optimality orthogonal matching pursuit (DOOMP) for skin lesions utilizing a fixed wavelet grid network (FWGN). The method achieved an accuracy rate of 91.8%.

### 2.6. Deep Neural Network for Fuzzy Automatic Melanoma Diagnosis

Abbes et al. [24] suggested a model based on the ABCD rule for feature extraction, fuzzy c-means (FCM) to estimate membership degree, and a deep neural network classifier for decision making. As a consequence, the model achieved an accuracy of 87.5%.

### 2.7. Automatic Melanoma Detection via Multi-Scale Lesion-Biased Representation and Joint Reverse Classification

Bi et al. [25] suggested an automated melanoma identification approach for dermoscopic images based on multi-scale lesion-biased representation (MLR) and joint reverse classification (JRC). For classification, the JRC model was utilized, and it gave extra facts for melanoma detection. The suggested technique was evaluated and tested using the PH2 public database.

### 2.8. Improving Dermoscopic Image Segmentation with Enhanced Convolutional Deconvolutional Networks

Yuan and Lo [26] presented deep fully convolutional deconvolutional neural networks (CDNNs) to build binary masks for skin lesion segmentation in dermoscopic photos. The input picture was filtered into skin and lesion classes using pixel-by-pixel classification. The training procedure was utilized to minimize a loss function based on the Jaccard distance. Each CDNN had 29 layers, and its hyper-parameters were determined via grid search. To restore picture resolutions, upsampling and deconvolutional layers were applied. For the segmentation challenges, RGB, hue–saturation–value (HSV), and lighting in LAB regions were taken into account. The final segmentation output was obtained using an ensemble model with six CDNNs as a base classifier. Their research demonstrated greater resilience for lesion segmentation.

### 2.9. Production of the Grounds for Melanoma Classification Using Adaptive Fuzzy Inference Neural Network

In 2013, Ikuma and Lyatomi [27] developed a new melanoma detection screening method based on the adaptive fuzzy inference neural network (AFINN), in which, just 88 fuzzy rules were established, resulting in 88 nodes in the AFINN’s rule layer. The model has a sensitivity (SE) of 81.5% and a specificity (SP) of 73.9%, although it is inferior to other multi-layer neural networks or non-linear models because of its restricted classification capacity.

### 2.10. Event-Triggered Control for Robust Set Stabilization of Logical Control Networks

Li and Shen [28] suggested a deep learning architecture for lesion segmentation and coarse lesion classification that comprises two fully convolutional residual networks (FCRN). The categorization findings were enhanced further by using a lesion index calculation unit (LICU) that calculated the relevance of pixels depending on their proximity to the adjacent boundary. To build the first coarse maps, the two FCRNs were trained on the basic model and also on flipped and rotated augmented pictures. The modified maps were created by convolving the distance maps generated by the LICU with the coarse maps. The final lesion classification findings were based on the average probability of the improved maps.

### 2.11. Automated Skin Lesion Analysis Based on Color and Shape Geometry Feature Set for Melanoma Early Detection and Prevention

Abuzaghleh et al. [29] suggested a method for an enhanced diagnosis and prevention of melanoma depending on the color and shape geometry extracted features from the examination of skin lesions. SVM was implemented as a classifier at both levels, with a single classifier in the first level and dual classifiers in the second level. The research claimed that the two-level classifier beat the single-level classifier, implying that two-level classifiers are more resilient than single-level classifiers. A multi-level classification job, as presented in this research, inevitably slows down the model’s calculation performance, proving to be a severe flaw.

### 2.12. Skin Lesion Classification Using Deep Multi-Scale Convolutional Neural Networks

DeVries and Ramachandram [30] used a multiscale Inception-v3 network to classify skin lesions. The model was trained on ImageNet and fine-tuned with the ISIC 2017 dataset utilizing two alternative picture resolutions, namely low resolution and high resolution centre-crop photos. The collected features were synthesized and sent on to the fully connected layer for both resolutions. The weights of the last two inception blocks and the fully connected layers were changed during the training phase, but all other layers in the network remained frozen. For each test image, augmented images were created for evaluation. A network was created as a consequence, with several training choices. As the final classification output, the combined result of these variation deep networks was adopted.

## 3. Materials and Methods

**Figure 2:**
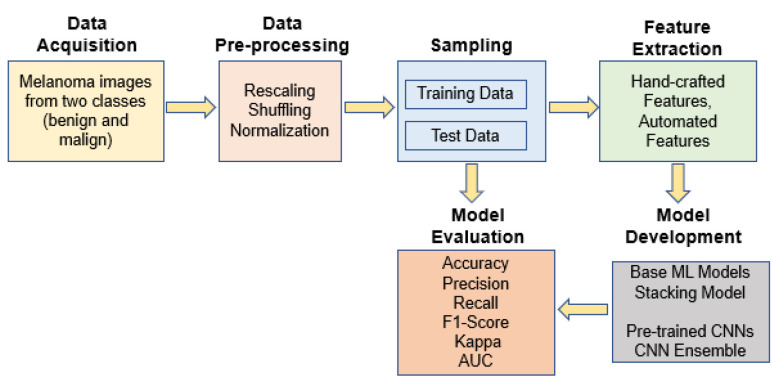
The overall design of the proposed system for melanoma skin cancer detection.

The overall system design for diagnosing melanoma skin cancer is depicted in Figure 2 as a series of steps. First, input melanoma images were pre-processed to enhance their quality. The pre-processed data set was then divided into two groups: training and testing. Thereafter, handcrafted features based on shape, texture and color were used to train the base machine learning models and the stacked ensemble model. In addition, automated features were extracted using fine-tuned CNN models pre-trained on an ImageNet dataset. The prediction results from the best models were then ensembled to increase the performance of detecting the disease. The remainder of the section details each phase of the proposed system.

### 3.1. Dataset Description and Pre-Processing

A small dataset [10] of the ISIC 2018 [11] challenge dataset was used to build a classifier to process an image and classify skin lesion classes. Dermoscopic images from any disease category except melanoma were excluded while curating the small dataset used in this study. Since the original dataset of skin lesions is highly imbalanced, this small dataset provides a balanced dataset of images containing two classes: benign skin moles and malignant skin moles. An equal number of images of both classes were selected randomly to be included in this curated dataset. The dataset includes a total of 3297 images (224 × 224). It already has a training and a test set. The training set has a total of 2637 images divided into two classes: benign class with 1440 images and malignant class with 1197 images. However, the test set has a total of 660 images with 360 benign skin mole images and 300 malignant skin mole images. For the classifier models proposed, the small dataset was mainly used, but the models can also classify the multi-class ISIC 2018 dataset. The class (either benign or malign) of the melanoma images is designated as the primary output variable of the study. Class values of zero and one indicate a benign and a malignant skin mole, respectively.

We applied various pre-processing tasks on the training and test images before model training and testing. Scaling data prior to training and testing deep learning models is critical. Input images that are not scaled properly can lead to a delayed and unsteady learning process. We used a scaling method called normalization to change the scale of the input images. To normalize, we converted input image data from their original values to a range of values between −1 and +1.

Additionally, we attempted to enhance the dataset’s quality and size by utilizing a variety of data augmentation approaches that have been successfully applied to increase performance in image classification challenges in medical disciplines [31]. Improving the training data size with augmented images will help us deal with the fact that we have a limited number of training images. Table 1 describes the augmentation approaches we used on our training dataset.

### 3.2. Model Architecture

Generally, skin lesions are taken lightly, and they are already in their final stage by the time a person realizes them. Hence, detecting malignant skin moles in the early stage is essential. Both deep learning and machine learning models play an important role in classifying skin lesions. To this end, two model architectures were proposed in our study: (i) using classical machine learning models with “hand-crafted” images features, where the final prediction was made by training the level 1 model stacking using 5-fold cross-validation on training set from the predictions made by the base machine learning models, and (ii) transfer learning by fine-tuning the backbones that are already trained on ImageNet dataset, and then using deep learning models to make predictions, followed by ensembling different combination of best models to achieve a better accuracy result.

#### 3.2.1. Classical Machine Learning Models with “Hand Crafted” Image Features

**Figure 3:**
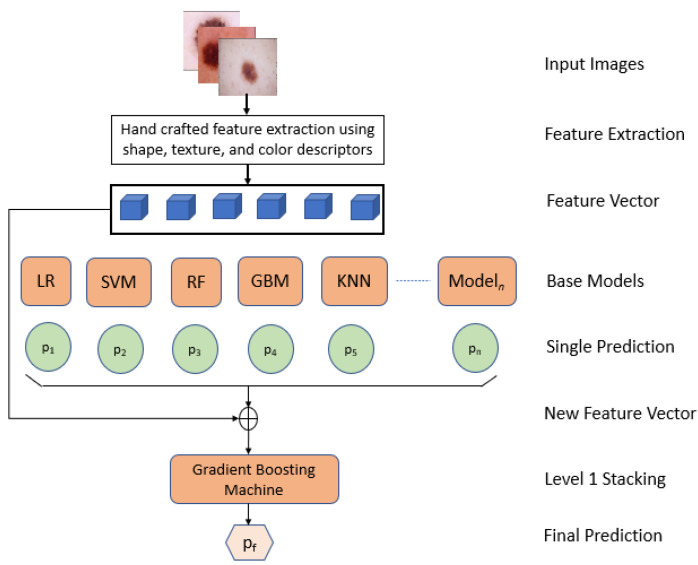
Stacking ensemble of machine learning models with “hand-crafted” image features.

At first, feature extractions were performed by calculating the global shape, texture, local keypoint descriptors, and color descriptors for the images using Hu moments, Haralick features, and color histogram. Hu moments are moments that are not affected by translation, scaling, or rotation. As a result, Hu moments are utilized in the form matching process. Haralick texture features are calculated using a gray level co-occurrence matrix (GLCM), which is a matrix that calculates the co-occurrence of adjacent gray levels in an image. It is a square matrix with a dimension equal to the number of gray levels N in the target area (ROI). Most of these descriptors are translation, scale, and rotational invariant. That means that the translation, scale, and rotation of the images cannot be changed. The machine learning base models were then trained with different hyper-parameters using these “hand crafted” features. As shown in Figure 3, the hand-crafted feature extraction was fed into the base models. A total of 49 classifiers with different hyper parameters were produced from the base models. Each model was then evaluated, and the metrics of accuracy, f1-score, Cohen’s kappa, and confusion matrix were calculated. The machine-learning-based models that were used are gradient boosting machine, KNN, logistic regression, random forest, and SVM. The predictions made by each of the 49 models were then added to the feature vector. Then, level 1 model stacking was trained using 5-fold cross-validation on training set, and a final prediction was recorded as an average calculated from all of the folds. The stacking resulted in improved accuracy, f1-score, and Cohen’s kappa compared to the other models.

#### 3.2.2. Deep Learning and Transfer Learning Followed by Ensembling

**Figure 4:**
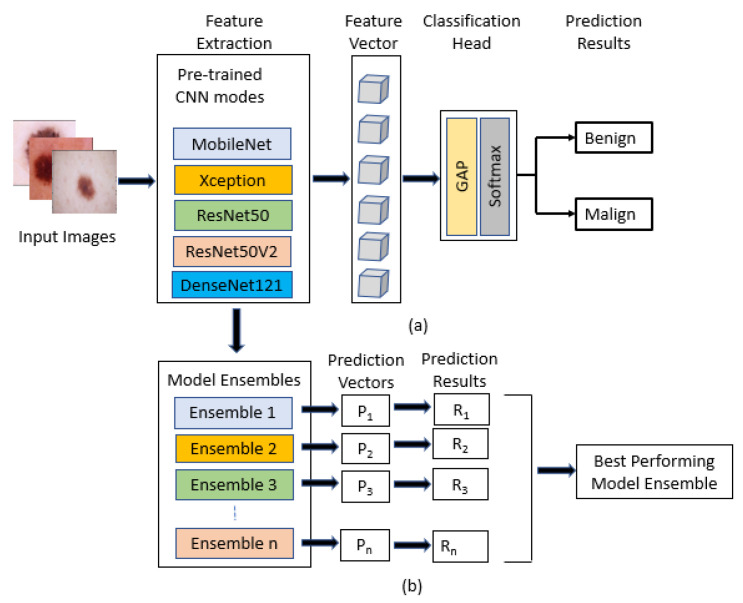
Deep learning models for melanoma skin cancer detection: (**a**) schematic diagram of modified pre-trained CNN models, (**b**) ensemble models to produce an optimal predictive model.

Convolutional neural networks and deep learning models are becoming the first choice to detect skin diseases because of the accuracy and perfection gained by the models. The feature parameters are shared in CNN models, and due to dimensionality reduction, they yield less computation time. Here, instead of a “hand-crafted” feature, the method proposes a convolutional neural network to learn the feature representation of images. Transfer learning concept was used by fine-tuning the backbones, since the photos used to train the models are not very large. It has a total of 2637 training set images. For transfer learning, five models were used: ResNet50, ResNet50V2, Mobilenet, Xception, and DenseNet121, which are already pre-trained on ImageNet data. After fine-tuning the backbones, the top fully connected layer of these pre-trained models was removed. A global average pooling and a new classification head with softmax activation were then attached, as shown in Figure 4. Global average pooling was used to reduce the number of model parameters without affecting the prediction result. First, the weights of the backbones were frozen to only train the classification head with softmax activation, and then all layers were trained to fine-tune the models. The images in the dataset have different scales. As the convolution operation is translation invariant, image augmentation was carried out during the training to help the model with a better generalization. All five models were then predicted and evaluated. Figure 4 shows that the prediction of the best models were used in different combinations to ensemble them to produce one optimal predictive model. Then, the ensembled models were evaluated and their accuracy, f1-score, Cohen’s, kappa, and confusion matrix were recorded. The next section elaborately describes the results of these models and compares them to find out the best classifier method.

### 3.3. Statistical Analysis

Our dataset contains skin lesion images from two categories, namely, benign skin moles and malignant skin moles. A total of 3297 images were sampled from the original ISIC 2018 challenge dataset from melanoma disease only. We used stratified probability sampling or randomization method to choose the images, where the samples were drawn for each stratum (group) at random but in proportional allocation. Furthermore, we adopted an unblinded technique, where randomization cannot be concealed.

As part of the statistical hypothesis testing, we used the corrected paired Student’s *t*-test to see whether the differences in the performances of various studied models are statistically significant or not. To accomplish this, the paired *t*-test was utilized using the null and alternative hypotheses stipulated earlier. Statistical significance was determined by *p* values less than 0.05. We made the following assumptions to use this method of testing regardless of whether there are significant differences between two sets of data. The measurements taken for one subject have no effect on the measurements taken for any other subject, and vice versa. Any of the paired measures must be acquired from the same subject. Finally, the observed performance differences follow a normal distribution. Both quantitative and qualitative results were provided to assess the effectiveness of the proposed models in detecting melanoma skin cancer in new patients. Among the quantitative data, accuracy and Cohen’s kappa are the most prominent. Qualitative results were supported by the suggested interpretability approach, which generates heatmaps to identify the parts of a melanoma image that are most suggestive of the disease. We used SciPy (1.8.0), which is a Python library for scientific computing, and is available for free and open source use.

## 4. Results and Discussions

In this paper, two methods were proposed. The first method includes the machine learning models followed by level one stacking, and the second method comprises deep learning models followed by ensembling. For both approaches, we used the miniature version of the ISIC 2018 challenge dataset. The two classes of the dataset were balanced, and it already contained the training set and test set. To compare the models, we evaluated the models with different metrics. These metrics include: accuracy—which measures the proportion of correct predictions, ranging from 0 (worst) to 1 (best); f1-score—the harmonic mean of recall and precision, which measures from 0 (worst) to 1 (best); Cohen’s kappa—measures how agreeable the true label and prediction are, ranging from −1 (completely disagree) to 1 (completely agree); confusion matrix—an overview of prediction results on a classification problem. AUC-ROC curves of the models were also generated to provide a performance measurement at various threshold settings. In fact, ROC curves are widely used to graphically depict the relationship/trade-off between clinical sensitivity and specificity for every conceivable cut-off for a model or a group of models. ROC curves are used to determine the best cut-off value for a model. The most suitable cut-off has a high true positive rate while having a low false positive rate and is determined on the ROC curve that is closest to the graph’s upper left corner. A single measure, the area under the ROC curve (AUC), may summarise all potential configurations of sensitivity and specificity that could be produced by adjusting the cutoff value. Thus, the AUC is a measure of how accurate a model is. The greater the AUC, the more accurate the model. When comparing two models, an AUC greater than or equal to the other’s is a sign that the model with the higher ROC curve is the more accurate of the two. Thus, we have used area under the curves values (AUC) to compare the performance of the deep learning models. The GPU and TPU support from Google Colab were used, and the models were trained. Table 2 describes our model parameters and setup.

Table 3 and Table 4 show the result of our first method. A total of 49 classifiers were produced with different hyper parameters from the five base models: logistic regression, random forest, support vector machine, gradient boosting machine and k-nearest neighbor. The best result of each model are recorded. From the following table, it can be seen that the k-nearest neighbor performed last, with an accuracy of 82%, f1-score of 83%, and Cohen’s kappa of 64%. Logistic regression and random forest performed slightly better, with an accuracy and f1-score of 84%, and Cohen’s kappa of 69% and 68%, respectively. The support vector machine performed better than the other three models, with an accuracy and f1-score of 85% and Cohen’s kappa of 69%. Among the base models, the gradient boosting machine performed well, with an accuracy of 87%, f1-score of 87%, and Cohen’s kappa of 74%. We then performed level one stacking on the feature vector where the predictions of all the 49 classifiers were stored, and calculated the final prediction result. After level one stacking, the accuracy rose to 88%, f1-score rose to 88%, and Cohen’s kappa rose to 76%.

While training the deep learning models, the hyper-parameters were tuned with random search. The classification head and all layers were trained up to 100 epochs separately. The batch size and learning rate were set to 32 and 0.001, respectively. The training was halted when the model had no improvements, and the accuracy curve started to converge to a specific limit. The table shows the evaluated results of the models. In TPU, the classification head, on average, took up to 6 min for each epoch; on the other hand, to train all layers, it took an average of 16 min for each epoch. Therefore, if the classifier trained its classification head for 100 epochs and then all layers for another 100 epochs, then the total training time for the classification head would be 600 min and, for all layers, 1600 min, which means that the classifier would take around 2200 min to train in total.

**Figure 5:**
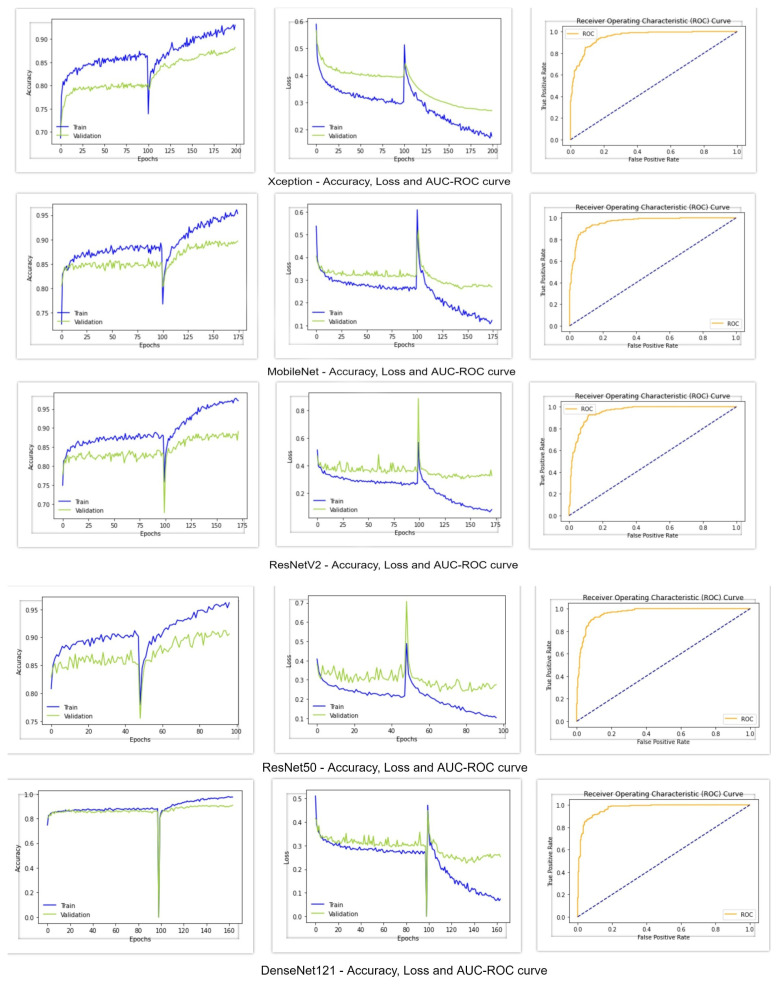
Deep learning models—accuracy, loss, and AUC-ROC curve.

Five deep learning models—MobileNet, Xception, ResNet50, ResNet50V2, and DenseNet121—were pre-trained on ImageNet data, and transfer learning was used by fine-tuning them. When we fed our training data into these classifier models, the following results, shown in Table 5 and Table 6, were obtained. Figure 5 shows the accuracy, loss, and AUC-ROC curve of these deep learning classifiers. From the following table, it can be seen that the Xception architecture performed poorly compared to the other deep learning models, with an accuracy and f1-score of 88%, Cohen’s kappa of 77%, and AUC value of 0.95. On the other hand, all of the other deep learning classifiers achieved an accuracy greater than 90%. MobileNet and ResNet50V2 performed almost similarly, with an accuracy of 90%, f1-score of 90% and Cohen’s kappa of around 80%. However, MobileNet achieved an AUC value of 0.96, whereas the AUC value of ResNet50V2 was 0.95. Regarding the AUC-ROC curve of MobileNet and ResNet50V2, the curve of MobileNet is more rounded at the edges. ResNet50 performed better than the other models, with an accuracy of 91%, f1-score of 91%, Cohen’s kappa of 82%, and AUC of 0.96. DesnetNet121 achieved an accuracy of 91%, f1-score of 90.8%, Cohen’s kappa of 81.7%, and AUC of 0.97, which is better than all of the other base models. The accuracy and loss curves of all of the models in Figure 5 show a region where there is a massive change in the middle of the curves. The portion of the curve before the massive change shows the training of the classification head of the classifiers, and the portion after the massive change shows the training of all of the other layers of the classifiers. Since the classification head was first trained, and reached a certain accuracy and loss value, when the classifiers shifted to train all of their layers, a massive change in results occurred. From these graphs, it can be seen that the classification head training pushed the accuracy to a certain level and, after training all layers, it further pushed the accuracy to its maximum level.

**Figure 6:**
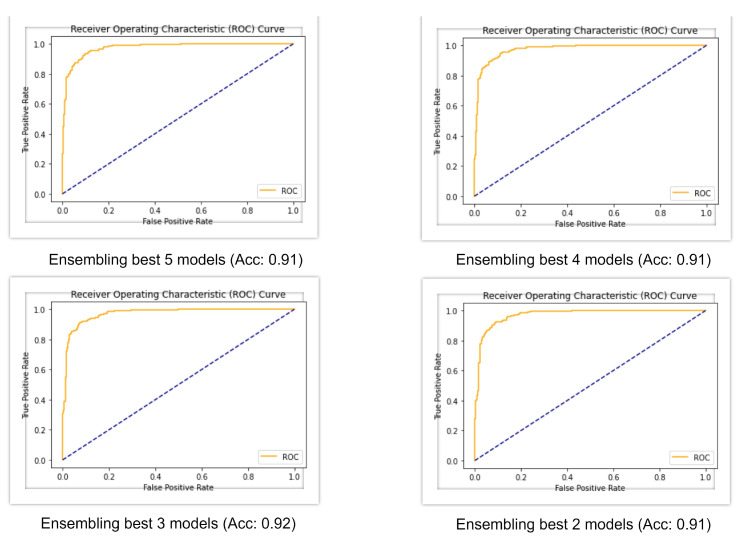
AUC-ROC curve of ensembled models.

The best base models of deep learning were then ensembled with combinations of five, four, three, and two. Figure 6 shows the AUC-ROC curve for four ensembled models and, from the table, it can be seen that when all the five models were ensembled, it gave an accuracy of 91%, f1-score of 91%, Cohen’s kappa of 82%, and AUC of 0.97. When the four best models were ensembled, they produced a similar result, but when the three best models were ensembled, the accuracy rose to 92%, f1-score rose to 92%, and Cohen’s kappa rose to 83%, which is greater than all the machine learning and deep learning models. All of the ensembled models produced an AUC value of 0.97.

From the above results, we can compare the models of machine learning and deep learning to classify skin lesions. Almost all the deep learning models showed an accuracy greater than 90%, whereas Xception only achieved 88%. On the other hand, all of the machine learning models showed an accuracy below 90%. After stacking and ensembling, the final results are 88% and 92%, respectively. It can be clearly seen that, among all of the other methods, the ensembling of tthe op three deep learning models performed better, with an accuracy of 92%.

To measure the degree of significance in the difference between the performance of the best ensembling model and other CNN models, the *t* statistic as generated in [7] is combined with a Student-t distribution with a certain degree of freedom. A *p*-value below the accepted significance level (i.e., 5%) rejects the null hypothesis, affirming that the models’ performances are different. Furthermore, the null hypothesis will not be rejected if the *p*-value is higher than the significance level. We employed this approach to compare the performance metrics of the best ensembling model with each of the base models by computing the paired *t*-test results, as shown in Table 7. The *p*-values smaller than the considered threshold level (i.e., 5%) are highlighted in bold, implying that the null hypothesis can be rejected, and the studied models perform differently in such cases. Therefore, the findings statistically show convincing evidence that the performance gain of the ensembling model, especially in terms of the kappa, accuracy, and AUC values over the base models, is significant.

The ISIC 2018 challenge winner MetaOptima [32], achieved an accuracy of 0.871 on the best single model, 0.882 on the meta ensemble, and 0.885 on the top 10 models averaged. This means that our proposed model’s accuracy is almost 4% greater than that of the ISIC 2018 challenge winners. We also compare the performance of our best performing model with some other existing work from the literature. A comparative summary of these techniques is provided in Table 8. Due to the fact that different research used distinct datasets and performance indicators, it would be difficult to carry out an effective comparison. Table 8 shows that our model outperforms many other methods when they use standard datasets. When compared to studies that used the ISIC 2018 dataset, our model outperforms others in terms of accuracy (92.0%), precision (91.0%), and sensitivity (92.0%).

Furthermore, the suggested ensemble model has a high AUC score (0.970), indicating that our approach can appropriately identify the population with melanoma skin cancer problems. Our study is surpassed in terms of all metrics by the models reported by Yuan and Lo [26], who assessed their models using datasets other than ours. However, the majority of this research in the literature does not provide any or sufficient explanation for their performance results. Our model, on the other hand, gives results that are important for AI models to be accepted by a wide range of health care professionals.

**Figure 7:**
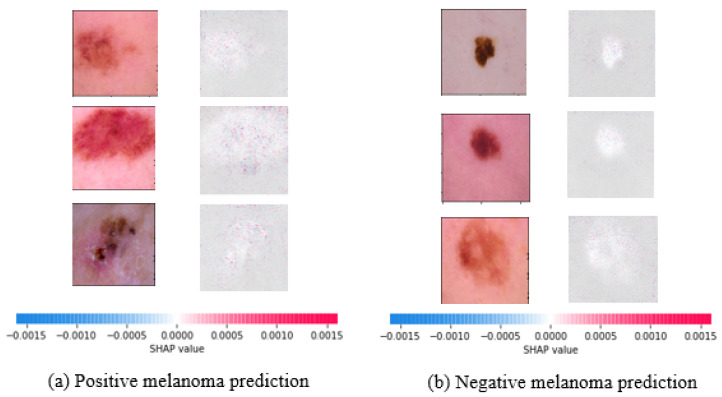
Interpretation of prediction results for both positive and negative melanoma predictions using the singleton best performing DenseNet121 model.

To this end, SHAP values for the best performing DenseNet121 model are shown in Figure 7. Indicators in red reflect features that increase the malignant melanoma output, whereas indicators in blue show features that decrease it. The saliency of a ROI is measured by the sum of the intensity of its features for a given class. As a result, the robustness of our best performing deep learning strategy for malignant melanoma detection is explained by this explainability strategy.

We can understand how AI can help dermatologists to diagnose potential melanoma skin cancer difficulties more correctly and rapidly, given the amount of effort made so far to employ deep learning models to autonomously assess the severity of the disease. This research takes us closer to a better understanding of the problems that skin cancer causes. In comparison to other studies in the area, it offers an improved deep-learning-based strategy for the automatic and quick detection of prospective skin cancer occurrences.

Our proposed fusion approach, nonetheless, is not intended to replace the knowledge of a dermatologist. Rather, we think that our findings will have a big impact on how AI-assisted technologies are used in the medical field. Health care providers can greatly benefit from automatic advanced scanning to discover positive encounters while waiting for more comprehensive testing to be specified, even if they cannot solely rely on the outcomes of skin lesion images to advise a patient’s treatment plan.

## 5. Conclusions and Future Work

Artificial intelligence techniques are now extensively used in modern medical diagnosis systems. Skin lesion detection is essential in the early stages, and providing an automatic diagnosis system that detects cancerous cells will save many lives. In this paper, we introduced an interpretable approach to melanoma skin cancer diagnosis based on deep learning and ensemble stacking of machine learning models. According to the results, deep-learning-based methods proved to be more reliable and dominant. The highest ranked ensemble model obtains an excellent accuracy (92.0%) and AUC score (0.97) for identifying melanoma with a high degree of precision and recall. Since the training was carried out on a small dataset with only two classes, the methods can also classify the large dataset. As the classes are highly imbalanced, the small dataset provided more balanced classes. The main obstacle faced during building a skin lesion classifier is not having a large balanced public dataset. In addition, images contain occlusions, such as hair or a ruler. Furthermore, the findings of our experiments suggest that our model exhibits good interpretability features by accurately recognizing a variety of melanoma-related symptoms.

A method will be proposed in future work, where the machine learning and deep learning approaches will be combined to test the classifier’s effectiveness. A more efficient data purification method will be identified to make the images occlusion-free. Finally, by having a better data purification method and a large balanced dataset with the proposed future method, the classifier’s accuracy can be pushed further.

## Figures and Tables

**Table 1:** Approaches for image augmentation in training dataset.

Method	Amount
rotation_range	90
shear_range	0.1
zoom_range	0.1
horizontal_flip	True
vertical_flip	True
shuffle	True

**Table 2:** Summary of model parameters and configurations.

Item Description	Value
Epoch Count	100
Batch Size	16
Type of Optimizer	SGD with a Nesterov momentum of 0.9
Learning Rate	Initial rate of 0.001 with a decay of 1 × $10^{-6}$
Loss Function	Binary Cross Entropy
Image Size	Both 299 × 299 and 224 × 224
Pooling Technique	Global Average Pooling (GAP)
Activation Function	Softmax (Classification Head)
Pre-trained Weight	ImageNet

**Table 3:** Performance report of method 1.

Techniques	Accuracy	Precision	Recall	F1-Score	Kappa
Logistic Regression	0.84	0.83	0.87	0.84	0.69
Random Forest	0.84	0.75	0.93	0.84	0.68
SVM	0.85	0.81	0.89	0.85	0.69
GBM	0.87	0.83	0.91	0.87	0.74
KNN	0.82	0.88	0.80	0.83	0.64
Stacking	0.88	0.84	0.92	0.88	0.76

**Table 4:** Confusion matrix of method 1.

Techniques	TP	FP	FN	TN
Logistic Regression	299	61	42	258
Random Forest	270	90	18	282
SVM	292	68	33	267
GBM	302	58	28	272
KNN	319	41	76	224
Stacking	305	55	26	274

**Table 5:** Performance report of method 2.

Techniques	Accuracy	Precision	Recall	F1-Score	Kappa	AUC
Mobilenet	0.90	0.89	0.92	0.90	0.80	0.96
Xception	0.88	0.91	0.87	0.88	0.77	0.95
ResNet50	0.91	0.91	0.91	0.91	0.82	0.96
ResNet50V2	0.90	0.88	0.93	0.90	0.80	0.95
DenseNet121	0.91	0.90	0.92	0.91	0.82	0.97
Ensembling (5 models)	0.91	0.91	0.92	0.91	0.82	0.97
Ensembling (4 best models)	0.91	0.90	0.92	0.91	0.82	0.97
Ensembling (3 best models)	0.92	0.91	0.92	0.92	0.83	0.97

**Table 6:** Confusion matrix of method 2.

Techniques	TP	FP	FN	TN
Mobilenet	321	39	27	273
Xception	329	31	45	255
ResNet50	331	29	30	270
ResNet50V2	317	43	23	277
DenseNet121	327	33	27	273
Ensembling (5 models)	329	31	27	273
Ensembling (4 best models)	327	33	26	274
Ensembling (3 best models)	331	29	26	274

**Table 7:** Paired *t*-test results (*t*-test stat, *p*-value) from the performance comparison of the best ensembling model and other base CNN models.

Techniques	Accuracy	Precision	Recall	AUC	Kappa
Mobilenet	(**−2.954**, **0.014**)	(**−2.583**, **0.029**)	(−1.507, 0.182)	(**−2.872, 0.016**)	(**−3.911, 0.002**)
Xception	(**−3.460**, **0.006**)	(−1.280, 0.229)	(**−4.682**, **8 × $10^{-4}$**)	(**−5.230**, **3 × $10^{-4}$**)	(**−5.140**, **4 × $10^{-4}$**)
ResNet50	(**−2.859**, **0.016**)	(−1.280, 0.229)	(**−2.859**, **0.016**)	(**−2.572**, **0.027**)	(**−2.230, 0.049**)
DenseNet121	(**−2.859**, **0.016**)	(**−2.269**, **0.049**)	(−1.441, 0.199)	(**1.347**, **0.207**)	(**−2.230, 0.049**)

**Table 8:** A comparative summary of the existing techniques for skin lesion classification.

Research Team	Technique	Dataset	Results
Devansh et al. [12]	De-coupled DCGANs	ISIC-2017 dataset	ROC-AUC 0.880, Accuracy 81.6%
Vijayalakshmi [14]	Convolutional neural network (CNN) and support vector machine (SVM)	ISIC 2018 dataset	Accuracy 85.0%
Daghrir et al. [15]	CNN and two machine learning classifiers (KNN and SVM)	ISIC 2018 dataset	Accuracy 88.4%
Nasr et al. [19]	Custom CNN model	MED-NODE dataset consisting of 170 images (70 melanoma and 100 nevi cases)	Accuracy 81.0%, Precision 75.0%, Sensitivity 81.0%
Warsi et al. [23]	Segmentation and classification approach based on D-optimality orthogonal matching pursuit (DOOMP), fixed wavelet grid network (FWGN)	PH2 dataset	Accuracy 91.8%, Specificity 92.5%
Abbes et al. [24]	Fuzzy c-means (FCM), deep neural network	A public dataset consisting of 206 lesion images	Accuracy 87.5%, Sensitivity 90.1%, Specificity 84.4%
Bi et al. [25]	Multi-scale lesion-biased representation (MLR) and joint reverse classification (JRC)	PH2 dataset	Accuracy 92.0%, Sensitivity 87.5%, Specificity 93.1%
Yuan and Lo [26]	Deep fully convolutional deconvolutional neural networks (CDNNs) to build binary masks for skin lesion segmentation	ISBI 2017 skin lesion dataset	Accuracy 93.4%, Jaccard Index (JA) of 0.765, Sensitivity 82.5%
Abuzaghleh et al. [29]	Skin lesion segmentation and analysis based on color and shape geometry, SVM classifier	PH2 dataset	One-level accuracy 91.0%, Two-level accuracy 93.2%
DeVries and Ramachandram [30]	Inception-V3 model	ISIC 2017 dataset	Accuracy 90.3%, AUC 0.943
Our approach	Interpretable deep learning and ensemble stacking of ML models	A small dataset of ISIC 2018	Accuracy 92.0%, Prec. 91.0%, Recall 92.0%, Kappa 0.83, AUC 0.97

## Data Availability

Not applicable.
